# Variables related to locoregional and distant recurrence in esophageal cancer

**DOI:** 10.1590/0102-67202025000020e1889

**Published:** 2025-08-04

**Authors:** Sarah FONSECA, Igor Gabriel Silva RAMOS, Felipe Antonio Boff MAEGAWA, Pedro Luiz Serrano USON, Francisco TUSTUMI

**Affiliations:** 1 Universidade de São Paulo, Department of Gastroenterology – São Paulo (SP), Brazil.; 2 Centro Universitário Lusíada, Department of Evidence-Based Medicine – Santos (SP), Brazil.; 3 Emory University, Department of Surgery, Atlanta (GA), USA.; 4 Hospital Israelita Albert Einstein, Center for Personalized Medicine – São Paulo (SP), Brazil.; 5 Mayo Clinic Cancer Center, Department of Oncology – Phoenix (AZ), USA.

**Keywords:** Esophageal Neoplasms, Recurrence, Prognosis, Surgical Procedures, Neoplasm Staging, Neoplasias Esofágicas, Recidiva, Prognóstico, Procedimentos Cirúrgicos, Estadiamento de Neoplasias

## Abstract

**Background:**

Esophageal cancer remains one of the most aggressive malignancies of the gastrointestinal tract, with high rates of recurrence and mortality despite curative-intent surgery and adjuvant therapies. Identifying factors associated with recurrence is crucial for improving outcomes and guiding personalized treatment.

**Aims:**

The aim of this study was to evaluate pretreatment and treatment-related variables associated with recurrence in patients with esophageal cancer undergoing surgical resection.

**Methods:**

This retrospective study analyzed data from patients with stage I–III esophageal carcinoma who underwent esophagectomy between 2000 and 2025, using the Fundação Oncocentro de São Paulo (FOSP) database. Clinical, histological, and treatment-related variables were evaluated. Disease-free survival and recurrence patterns were assessed using Cox proportional hazards models and Fine–Gray subdistribution hazard models.

**Results:**

A total of 2,057 patients were included, with a mean follow-up of 36.5 months (±44.8). In the multivariate analysis, advanced tumor stage (stage II: HR 1.68, 95%CI 1.21–2.33; stage III: HR 3.23, 95%CI 2.29–4.56; both p<0.01), location (middle esophagus: HR 1.31, 95%CI 1.11–1.54; p=0.001; upper esophagus: HR 1.54, 95%CI 1.21–1.96; p<0.001), and histological subtype (rare histologies: HR 2.17, 95%CI 1.35–3.49; p=0.001) were associated with worse disease-free survival. Multimodal therapy improved disease-free survival (HR 0.40, 95%CI 0.24–0.66) in stage III tumors. Squamous cell carcinoma was independently associated with locoregional recurrence (SHR 1.52, 95%CI 1.05–2.20; p=0.027). For distant recurrence, squamous cell carcinoma showed a protective effect (SHR 0.52, 95%CI 0.31–0.88; p=0.015), while high tumor grade (grade II: SHR 3.65, 95%CI 1.98–6.72; p<0.001) was associated with an increased risk. Multimodal treatments influenced recurrence patterns but did not independently predict outcomes after adjustment.

**Conclusions:**

Tumor stage, location, and histology were strong predictors of disease-free survival after surgery for esophageal cancer. Histological subtypes significantly influenced recurrence patterns. Squamous cell carcinoma was associated with a higher risk of locoregional recurrence but a lower risk of distant metastasis compared to adenocarcinoma. Multimodal therapy demonstrated a protective effect in stage III disease.

## ARTICLE HIGHLIGHTS

- Tumor stage, location, and histology were strong predictors of disease-free survival after surgery for esophageal cancer.
- Histologic subtypes significantly influenced recurrence patterns.
- Squamous cell carcinoma is associated with a higher risk of locoregional recurrence but a lower risk of distant metastasis compared with adenocarcinoma.
- Multimodal therapy effectively reduces recurrence in stage III tumors, emphasizing the importance of stage-specific treatment strategies.

## CENTRAL MESSAGE

In this population-based study of Brazilian patients undergoing surgery for esophageal cancer, tumor stage, tumor location, and histological subtype emerged as key predictors of disease-free survival. Squamous cell carcinoma was linked to an increased risk of locoregional recurrence but a reduced risk of distant metastasis compared to adenocarcinoma. Importantly, multimodal therapy significantly improved outcomes in patients with stage III disease.

## PERSPECTIVES

This study helps doctors to better manage patients after surgery for esophageal cancer. Identifying who is most at risk for cancer returning, based on preoperative information, supports more personalized follow-up and treatment plans. The findings also suggest that doctors should carefully consider when to use treatments such as chemo or radiation, especially in patients with more aggressive cancers. This can lead to better care and outcomes for those facing this severe disease.

## VISUAL ABSTRACT

**Figure:**
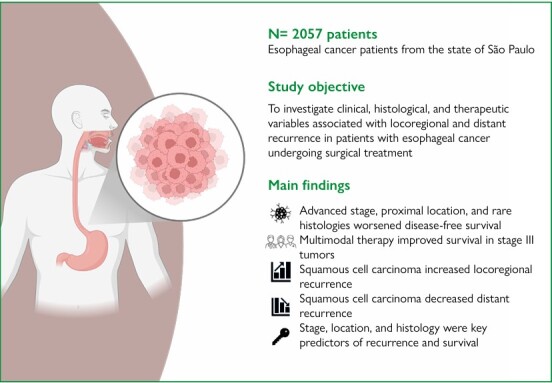

## RESUMO

**Racional:**

O câncer de esôfago continua sendo uma das neoplasias malignas mais agressivas do trato gastrointestinal, com taxas elevadas de recorrência e mortalidade, apesar da cirurgia com intenção curativa e das terapias adjuvantes. A identificação dos fatores associados à recorrência é crucial para melhorar os desfechos e orientar o tratamento personalizado.

**Objetivos:**

Avaliar as variáveis pré-tratamento e relacionadas ao tratamento associadas à recorrência, em portadores de câncer de esôfago, submetidos à ressecção cirúrgica.

**Métodos:**

Estudo retrospectivo que analisou dados de pacientes com carcinoma de esôfago em estágio I-III, submetidos à esofagectomia entre 2000 e 2025, utilizando o banco de dados da Fundação Oncocentro de São Paulo (FOSP). Variáveis clínicas, histológicas e relacionadas ao tratamento foram avaliadas. A sobrevida livre de doença e os padrões de recorrência foram avaliados utilizando modelos de riscos proporcionais de Cox e modelos de risco de subdistribuição de Fine-Gray.

**Resultados:**

O total de 2.057 pacientes foram incluídos, com um acompanhamento médio de 36,5 meses (±44,8). Na análise multivariada, o estágio avançado do tumor (estágio II: HR 1,68, IC95% 1,21–2,33; estágio III: HR 3,23, IC95% 2,29–4,56; ambos p<0,01), localização (esôfago médio: HR 1,31, IC95% 1,11–1,54; p=0,001; esôfago superior: HR 1,54, IC95% 1,21–1,96; p<0,001) e subtipo histológico (histologias raras: HR 2,17, IC95% 1,35–3,49; p=0,001) foram associados a pior sobrevida livre de doença. A terapia multimodal melhorou a sobrevida livre de doença (HR 0,40, IC95% 0,24–0,66) em tumores em estágio III. O carcinoma de células escamosas foi independentemente associado à recidiva locorregional (SHR 1,52, IC95% 1,05–2,20; p=0,027). Para recidiva distante, o carcinoma de células escamosas mostrou um efeito protetor (SHR 0,52, IC95% 0,31–0,88; p=0,015), enquanto o alto grau do tumor (grau II: SHR 3,65, IC95% 1,98–6,72; p<0,001) foi associado a um risco aumentado. Os tratamentos multimodais influenciaram os padrões de recidiva, mas não previram os resultados de forma independente após o ajuste.

**Conclusões:**

O estágio, a localização e o tipo histológico do tumor foram fortes preditores de sobrevida livre de doença, após cirurgia para câncer de esôfago. Os subtipos histológicos influenciaram significativamente os padrões de recorrência. O carcinoma de células escamosas foi associado a um risco maior de recorrência locorregional, mas a um risco menor de metástase à distância em comparação ao adenocarcinoma. A terapia multimodal demonstrou efeito protetor no estágio III da doença.

## INTRODUCTION

 Esophageal cancer stands as a highly aggressive malignancy within the gastrointestinal tract, holding a significant position in global cancer incidence and mortality, with approximately 570,000 new cases and 510,000 deaths estimated annually^6^. Despite advancements in diagnostic and therapeutic modalities, survival rates for esophageal cancer remain disappointingly low^11^. A paramount clinical challenge lies in the elevated rate of tumor recurrence, which severely impacts patient prognosis and quality of life^14^. This recurrence can manifest as locoregional or distant metastases, and its incidence remains substantial for both adenocarcinoma and squamous cell carcinoma (SCC), even following multimodal treatment strategies^12^. 

 Indeed, the persistent issue of recurrence is highlighted in multiple studies. A comprehensive analysis involving a large population-based cohort reported a 5-year recurrence rate exceeding 40% in patients undergoing surgery for esophageal cancer^7^. A systematic review and meta-analysis of neoadjuvant therapy followed by surgery revealed that approximately 30–50% of patients experience recurrence within 3 years posttreatment^8^. These figures underscore the significant burden of recurrence on long-term survival. 

 Despite advancements in treatment, recurrence rates following curative-intent surgery for esophageal cancer have remained relatively stable over the years. A cohort study analyzing patients treated surgically across three consecutive time intervals found minimal variation in recurrence rates^5^. Initially, 34.1% of patients experienced recurrence (1987–1997), followed by a modest decline to 28.8% (1998–2003) and later a rise to 33.7% (2007–2015). These findings suggest that, despite evolving surgical and perioperative strategies, the risk of recurrence continues to pose a significant challenge in esophageal cancer management. 

 Consequently, integrating clinical and biological features into risk stratification models is essential to identify patients at higher risk of recurrence. This approach enables more personalized treatment planning, optimizes postoperative surveillance, and helps guide the selection of adjuvant therapies to improve long-term outcomes^14^. 

 The persistent and significant challenge of recurrence in esophageal cancer necessitates a comprehensive understanding of the variables associated with its occurrence. This study aims to investigate factors associated with locoregional and distant recurrence in esophageal cancer to facilitate more effective and personalized postoperative management for patients treated with curative intent. 

## METHODS

### Study design and data source

 This is a retrospective population-based study conducted using data from the Fundação Oncocentro de São Paulo (FOSP). This public database compiles detailed oncological information from referral hospitals across the state of São Paulo, Brazil. The database includes demographic, clinical, histological, and treatment-related data, as well as survival and follow-up outcomes, making it a robust source for population based cancer research. 

### Study Period

 The study period spanned from 2000 to 2025. 

### Inclusion Criteria

 Patients were eligible for inclusion if they had a histologically confirmed diagnosis of esophageal carcinoma, were classified as TNM stages I to III at the time of diagnosis, underwent surgical treatment (esophagectomy), had a minimum of 3 months of follow-up data available, and received treatment at a referral center registered in the FOSP database. 

### Exclusion criteria

 Patients were excluded if they had non-carcinomatous esophageal tumors (such as sarcomas, lymphomas, or melanomas), TNM stage IV disease, metastasis at diagnosis, or unknown staging, missing or incomplete follow-up data, or if they underwent palliative treatment or received non-surgical treatment. 

### Variables Assessed

 The main variables investigated in this study included pretreatment and treatment-related factors, such as patient age, sex, tumor location, histological subtype, stage, tumor grade, and treatment modality (surgery alone, surgery combined with chemoradiotherapy, or surgery combined with chemotherapy). 

### Outcomes

 The primary outcome of interest was disease-free survival and cancer recurrence, whether locoregional or distant. Recurrence was defined as the time interval from the date of surgery to the date of documented recurrence or last follow-up. 

### Statistical analysis

 All statistical analyses were performed using Stata version 18.0 (StataCorp, College Station, TX, USA). Descriptive statistics were used to summarize the baseline characteristics of the included patients. Categorical variables were expressed as absolute frequencies and percentages, and continuous variables were summarized using means and standard deviations. 

 Disease-free survival was defined as the time from surgery to the occurrence of recurrence or death. Survival analyses were compared with Cox proportional hazards models. Univariate Cox regression analyses were initially performed to identify variables associated with disease-free survival. Variables with clinical relevance or a p< 0.05 in the univariate analysis were included in multivariate models. Interaction terms between treatment and stage were also evaluated. A forest plot based on the adjusted Cox regression models was constructed to represent the main findings visually. 

 For analyses of locoregional and distant recurrence, competing risks regression models were used, considering death without recurrence as a competing event. Univariate FineGray subdistribution hazard models were first performed to assess potential predictors. Variables of interest were then included in multivariate competing risk models. Hazard ratios (HRs) and subdistribution HRs (SHRs) were reported, along with corresponding 95% confidence intervals (CIs). A two-sided p-value of less than 0.05 was considered statistically significant. 

### Ethical aspects

 As this study involved a retrospective analysis of anonymized and publicly available data, ethical approval and informed consent were not required. 

## RESULTS

### Baseline characteristics

 A total of 2,057 patients were included, with a mean follow-up time of 36.5 months (±44.8 months). Most patients were male (81.8%) and aged 65 years or younger (74.3%). SCC was the predominant histological type (76.4%), and the majority of tumors were located in the middle or lower esophagus (89.2%). At diagnosis, 83.8% of patients had stage II or III disease. Surgery combined with chemoradiotherapy (49.9%) or chemotherapy alone (14.5%) was the most common treatment approach, while 35.5% underwent surgery alone. A detailed breakdown of patient characteristics is provided in Table 1. 

**Table 1:** Baseline characteristics of the included patients.

	n	%
Total		
-	2,057	100
Age (years)		
≤65	1,529	74.33
>65	528	25.67
Sex		
Male	1,683	81.82
Female	374	18.18
Location		
Lower	491	41.36
Middle	568	47.85
Upper	128	10.78
Cancer type		
Adenocarcinoma	417	21.26
SCC	1,499	76.44
Other histologies	45	2.29
Stage		
I	334	16.24
II	807	39.23
III	916	44.53
T stage		
T1	350	17.05
T2	379	18.46
T3	1,002	48.81
T4	322	15.68
N stage		
N0	1,117	55.35
N1	790	39.15
N2	78	3.87
N3	33	1.64
Grade		
1	255	37.17
2	421	61.37
3	10	1.46
Treatment		
Surgery	652	35.53
Surgery + CRT	917	49.97
Surgery + CT	266	14.50

SCC: Squamous cell carcinoma; CRT: Chemoradiotherapy; CT: Chemotherapy.

### Disease-free survival

 In the univariate analysis (Table 2), male sex, tumors located in the middle or upper esophagus, SCC and other histological types, advanced stage, higher T and N stages, and multimodal treatment were significantly associated with worse disease-free survival (all p<0.05). 

**Table 2:** Cox regression for disease-free survival

Disease-free survival			
	HR	95%CI	p-value
Age (years)			
>65	ref.		0.077
≤65	0.90	0.81–1.01	
Sex			
Female	ref.	25.67	**0.010**
Male	1.19	1.04–1.35	
Location			
Lower	ref.		
Middle	1.30	1.13–1.49	**<0.001**
Upper	1.52	1.22–1.88	**<0.001**
Cancer type			
Adenocarcinoma	ref.		
SCC	1.29	1.14–1.47	**<0.001**
Other histologies	1.68	1.21–2.34	**0.002**
Stage			
I	ref.		
II	1.61	1.37–1.89	**<0.001**
III	2.36	2.01–2.75	**<0.001**
T stage		
T1	ref.		
T2	1.41	1.18–1.68	**<0.001**
T3	1.80	1.55–2.09	**<0.001**
T4	3.05	2.56–3.64	**<0.001**
N stage			
N0			
N1	1.32	1.19–1.46	**<0.001**
N2	1.31	1.00–1.71	0.054
N3	1.60	1.09–2.35	**0.016**
Grade			
1	ref.		
2	1.07	0.89–1.29	0.488
3	1.65	0.81–3.34	0.169
Treatment		
Surgery	ref.		
Surgery + CRT	1.36	1.21–1.52	**<0.001**
Surgery + CT	1.38	1.17–1.63	**<0.001**

HR: Hazard ratios; CI: confidence intervals; p-values: are presented for each variable. SCC: Squamous cell carcinoma; CRT: Chemoradiotherapy; CT: Chemotherapy.

Significant p-values are highlighted in bold.

**Figure 1:**
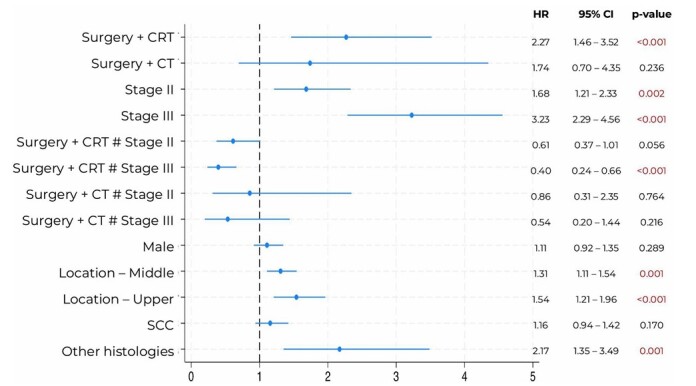
Multivariate Cox regression for disease-free survival.

 In the multivariate model (Figure 1), surgery followed by chemoradiotherapy (HR 2.27; 95%CI 1.46–3.52; p<0.001) and advanced stage (stage II: HR 1.68; p=0.002; stage III: HR 3.23; p<0.001) were independently associated with an increased risk of recurrence. However, patients with stage III disease treated with surgery plus CRT showed a significant reduction in recurrence risk (HR 0.40; 95%CI 0.24–0.66;p<0.001). Tumor location in the middle (HR 1.31; p=0.001) and upper esophagus (HR 1.54; p<0.001) and rare histologies (HR 2.17; p=0.001) were also independently associated with recurrence. 

### Locoregional recurrence

 In the univariate analysis, male sex (SHR 1.45; 95%CI 1.00–2.08; p=0.048) and SCC (SHR 1.35; 95%CI 0.961.90; p=0.081) showed a trend toward higher risk of locoregional recurrence. Other variables, including age, tumor location, stage, T stage, N stage, tumor grade, and type of treatment, were not found to be significantly associated with locoregional recurrence. 

 In the multivariate model, SCC remained independently associated with a higher risk of locoregional recurrence (SHR 1.52; 95%CI 1.05–2.20; p=0.027). No significant associations were observed for treatment type, sex, stage, or treatment with surgery plus chemotherapy after adjustment (Table 3). 

**Table 3:** Competing risk regression analysis for distant recurrence, considering death without recurrence as a competing event.

Locoregional recurrence						
	Univariate			Multivariate		
	SHR	95%CI	p-value	SHR	95%CI	p-value
Age (years)						
>65	ref.					
≤65	1.08	0.81–1.45	0.597			
Sex						
Female	ref.					
Male	1.45	1.00–2.08	**0.048**	1.27	0.87-1.86	0.210
Location						
Lower						
Middle	1.28	0.92–1.80	0.147			
Upper	0.90	0.49–1.64	0.721			
Cancer type						
Adenocarcinoma	ref.					
SCC	1.35	0.96–1.90	0.081	1.52	1.05-2.20	**0.027**
Other histologies	1.12	0.44–2.88	0.811	1.00	0.35-2.88	0.994
Stage						
I	ref.					
II	1.39	0.94–2.06	0.095	1.42	0.95-2.15	0.090
III	1.22	0.82–1.80	0.327	1.24	0.81–1.89	0.330
T stage*						
T1	ref.					
T2	1.44	0.95–2.19	0.088			
T3	1.26	0.87–1.83	0.217			
T4	0.97	0.60–1.57	0.913			
N stage*						
N0	ref.					
N1	1.08	0.84–1.39	0.555			
N2	0.70	0.31–1.59	0.396			
N3	0.50	0.12–2.03	0.334			
Grade†						
I	ref.					
II	0.78	0.48–1.27	0.321			
III	~0	0.00–0.00	**<0.001**			
Treatment						
Surgery	ref.					
Surgery + CRT	0.83	0.63–1.10	0.196	0.78	0.58-1.05	0.100
Surgery + CT	0.82	0.54–1.25	0.357	0.94	0.60–1.47	0.773
Distant recurrence						
	Univariate			Multivariate		
	SHR	95%CI	p-value	SHR	95%CI	p-value
Age (years)						
>65						
≤65	0.85	0.59–1.23	0.393			
Sex						
Female						
Male	0.94	0.61–1.43	0.769			
Location						
Lower						
Middle	0.94	0.63-1.39	0.748			
Upper	0.47	0.20–1.10	0.082			
Cancer type						
Adenocarcinoma						
SCC	0.65	0.45–0.95	**0.025**	0.52	0.31–0.88	**0.015**
Other histologies	1.18	0.46–3.03	0.726	0.55	0.13-2.31	0.414
Stage						
I						
II	2.05	1.11–3.79	**0.023**	1.14	0.50–2.59	0.756
III	1.98	1.07–3.66	**0.029**	1.27	0.56–2.87	0.572
T stage*						
T1						
T2	1.87	0.99–3.53	0.053			
T3	1.91	1.08–3.38	**0.026**			
T4	1.38	0.68–2.77	0.370			
N stage*						
N0						
N1	0.92	0.64–1.33	0.666			
N2	2.05	1.02–4.12	**0.044**			
N3	3.65	1.69–7.89	**0.001**			
Grade						
I						
II	4.13	2.20–7.77	**<0.001**	3.65	1.98–6.72	**<0.001**
III	5.21	1.11–24.43	0.036	4.75	0.97–23.29	0.055
Treatment						
Surgery						
Surgery + CRT	2.64	1.70–4.10	**<0.001**	1.65	0.83-3.27	0.154
Surgery + CT	1.58	0.84–2.99	0.159	0.49	0.17–1.37	0.173

SHR: Subhazard ratios; CI: confidence interval; p-values: are presented for each variable included in the model; SCC: Squamous cell carcinoma; CRT: Chemoradiotherapy; CT: Chemotherapy.

Significant p-values are highlighted in bold.

*Although T and N categories were evaluated in the univariate analysis, they were not included in the multivariable model because tumor stage (STAGE) already incorporates information on primary tumor (T) and nodal status (N). Including both would introduce collinearity and redundant adjustment, potentially biasing the estimates; †Although grade III tumors showed a statistically significant association in the univariate analysis, this subgroup had an extremely small sample size, leading to unstable estimates with implausible subhazard ratios. Therefore, the grade was not included in the multivariable model to avoid potential bias due to sparse data.

## DISCUSSION

 In this large study of patients undergoing surgery for esophageal cancer, tumor stage was the strongest independent predictor of disease-free survival, locoregional recurrence, and distant recurrence. Histological subtypes also significantly influenced recurrence patterns. SCC was associated with an increased risk of locoregional recurrence compared to adenocarcinoma, while it was independently associated with a lower risk of distant recurrence. 

 Staging remains a critical determinant of prognosis in esophageal cancer, as repeatedly demonstrated by the scientific literature^3^. Our findings reinforce the importance of early diagnosis and treatment, as patients with more advanced tumors were significantly more likely to experience recurrence. This association likely reflects the greater tumor burden and the presence of undetected micrometastases at the time of surgery, allowing microscopic disease to persist and manifest as relapse, particularly at locoregional sites^9^. 

 Importantly, our interaction analysis revealed that the benefit of multimodal therapy is stage-dependent. In patients with stage III disease, surgery combined with chemoradiotherapy significantly reduced the risk of recurrence compared to surgery alone. A similar trend, though not statistically significant, was observed in stage II disease. These results emphasize that the protective effect of combined therapy becomes particularly evident in advanced stages^8^. 

 Initially, in the unadjusted analysis, multimodal treatments appeared paradoxically associated with a higher risk of recurrence or death. This counterintuitive finding is likely due to indication bias, as patients with more advanced or biologically aggressive tumors are more frequently selected for multimodal therapies^4^. To address this potential confounding factor, an interaction term between treatment modality and tumor stage was introduced in the multivariable model. After adjustment, multimodal therapy emerged as a protective factor, particularly among patients with stage III disease. 

 Our findings demonstrated that neoadjuvant chemoradiotherapy followed by surgery improved survival and locoregional control in resectable esophageal cancer. Similarly, meta-analyses have confirmed that combined modality therapy provides a survival advantage, particularly in patients with advanced tumors^8^. These results underscore the necessity of tailoring treatment strategies according to tumor staging to maximize oncologic outcomes. 

 Although SCC and adenocarcinoma are the most common histological subtypes of esophageal cancer, rare histologies, grouped here as "other histologies," showed notably more aggressive behavior, as indicated by the higher HRs observed for disease-free survival in our multivariate analysis. This group includes entities such as adenosquamous carcinoma, neuroendocrine carcinoma, sarcomatoid carcinoma, and undifferentiated carcinomas, each characterized by distinct biological features associated with poor prognosis^12^. Previous studies have similarly reported poor survival rates and limited responsiveness to standard multimodal therapies in these subgroups, emphasizing the need for distinct therapeutic strategies and closer postoperative surveillance when such histologies are identified^11,12^. 

 Tumor location within the esophagus — upper, middle, or lower third — has important prognostic implications in esophageal cancer^10^. In our analysis, tumors located in the middle and upper esophagus were associated with a higher risk of recurrence and worse disease-free survival when compared to tumors of the lower esophagus. Several anatomical and biological factors can explain this observation. Tumors of the middle and upper esophagus are predominantly SCC, which tends to present with more extensive local invasion, early lymphatic spread due to the rich submucosal lymphatic network, and frequent invasion of critical mediastinal structures. Additionally, the complex lymphatic drainage of the upper and middle esophagus increases the likelihood of microscopic disease persistence even after complete surgical resection, contributing to higher rates of local and regional failure. Conversely, tumors of the lower esophagus, which are often adenocarcinomas, typically spread to a more predictable set of lymph nodes (such as celiac and perigastric nodes), making surgical clearance potentially more effective.^10^ Previous studies have similarly demonstrated that upper and middle esophageal tumors are associated with lower rates of R0 resection, higher rates of positive margins, and poorer overall survival compared to distal esophageal cancers^7,8^. 

 Cancer recurrence following esophagectomy may arise through various mechanisms and is generally categorized as either locoregional recurrence or distant metastasis. Locoregional recurrence refers to the reappearance of malignant disease within the esophageal bed, regional lymph nodes, or adjacent mediastinal structures. In contrast, distant recurrence is characterized by the hematogenous dissemination of tumor cells to remote organs, most commonly the liver, lungs, or bones. Locoregional recurrence typically manifests earlier in the postoperative course, likely due to residual microscopic disease left behind in the surgical field or surrounding lymphatic stations. Conversely, distant metastasis often reflects subclinical systemic spread that becomes evident only after a longer latency period^14^. 

 An interesting finding in our study was the differential impact of SCC histology on recurrence patterns. SCC was associated with a higher risk of locoregional recurrence but appeared to be a protective factor against distant metastasis. This observation may be explained by the biological behavior of SCC tumors, which tend to be more locally invasive, with early infiltration of adjacent mediastinal tissues and lymphatics, but with less frequent early hematogenous dissemination compared to adenocarcinomas. SCC is more often restricted to locoregional spread at initial recurrence. In contrast, adenocarcinomas, particularly those arising from Barrett’s esophagus at the gastroesophageal junction, demonstrate a higher propensity for distant metastasis early in the disease course^1,2^. This pattern aligns with prior evidence suggesting that histological subtype is a significant determinant of recurrence biology in esophageal cancer, reinforcing the importance of histology-specific risk stratification when planning postoperative surveillance and adjuvant therapy^13,14^. 

 This study has several limitations inherent to its retrospective, population-based design. Using registry data may introduce selection bias and is subject to variability in data quality and completeness across reporting institutions. Key clinical and pathological variables, such as resection margin status, lymphovascular invasion, and the exact number of metastatic lymph nodes, were unavailable in the dataset, limiting the ability to perform more granular risk stratification. Additionally, the long study period encompassed evolving treatment protocols and diagnostic criteria, contributing to therapeutic heterogeneity that may have influenced the outcomes. Despite these constraints, the large sample size and real-world scope offer valuable insights into recurrence patterns following esophagectomy. For future prospective, multicenter studies with standardized data collection are needed to validate these findings and enhance the precision of recurrence prediction and postoperative management strategies. 

## CONCLUSIONS

 Tumor stage, location, and histology were strong predictors of disease-free survival after surgery for esophageal cancer. Histological subtypes significantly influenced recurrence patterns. SCC was associated with a higher risk of locoregional recurrence but a lower risk of distant metastasis compared to adenocarcinoma. Multimodal therapy demonstrated a protective effect in stage III disease. 

## Data Availability

The Informations regarding the investigation, methodology and data analysis of the article are archived under the responsibility of the authors.
